# α-Amylase action on starch in chickpea flour following hydrothermal processing and different drying, cooling and storage conditions

**DOI:** 10.1016/j.carbpol.2021.117738

**Published:** 2021-05-01

**Authors:** Cathrina H. Edwards, Amalia S. Veerabahu, A. James Mason, Peter J. Butterworth, Peter R. Ellis

**Affiliations:** a King’s College London, Faculty of Life Sciences and Medicine, Departments of Biochemistry and Nutritional Sciences, Biopolymers Group, Franklin-Wilkins Building, 150 Stamford Street, London, SE1 9NH, United Kingdom; b King’s College London, School of Cancer & Pharmaceutical Science, Institute of Pharmaceutical Science, Franklin-Wilkins Building, 150 Stamford Street, London, SE1 9NH, United Kingdom

**Keywords:** ^13^C CP-MAS NMR, Cross Polarisation–Magic Angle Spinning Nuclear Magnetic Resonance, DMSO, dimethylsulphoxide, GC, gas chromatography, GI, glycaemic index, GL, glycaemic load, GLUT2, glucose transporter 2, HPLC, high-performance liquid chromatography, SGLT1, sodium-glucose cotransporter 1, PPA, porcine pancreatic α-amylase, PBS, phosphate buffer solution, RS, resistant starch, Starch digestion, Resistant starch, α-Amylase action, Log of slope analysis, Solid-state ^13^C CP-MS NMR

## Abstract

Starch is present in many prepared ‘ready-meals’ that have undergone processing and/or storage in frozen or chilled state. Hydrothermal processing greatly increases starch digestibility and postprandial glycaemia. Effects of different heating/drying and cooling regimes on amylolysis have received little attention. Hence, we examined the effects of different processing treatments on *in vitro* digestibility of starch in chickpea flour. Solid-state ^13^C NMR was used to estimate ordered double-helical structure in the starch. Native starch with 25 % double-helical content was the most resistant to digestion but hydrothermal processing (gelatinisation) resulted in >95 % loss of order and a large increase in starch digestibility. Air-drying of pre-treated flour produced slowly-digestible starch (*C*_∞_, 55.9 %). Refrigeration of gelatinised samples decreased ease of amylolysis coincident with increase in double-helical content. Freezing maintained the same degree of digestibility as freshly gelatinised material and produced negligible retrogradation. Chilling may be exploited to produce ready-meals with a lower glycaemic response.

## Highlights

• α-Amylase action on legume starch varied after different processing and storage regimes.
• Susceptibility to amylolysis linked to double-helical order as measured by ^13^C NMR.
• Chilling of gelatinised starch increased retrogradation and decreased digestion.
• Air-dried gelatinised starch markedly decreased the rate and extent of amylolysis.
• Various processing and storage strategies can lower starch digestion of ready-meals.

## Graphical abstract

**Figure:**
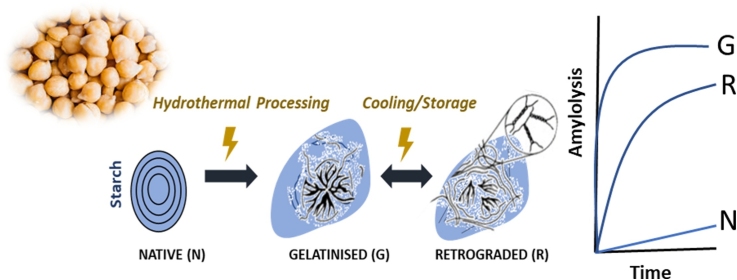

## 1 Introduction

Starch is a major source of exogenous glucose in humans, accounting for approximately 30 % or more of the UK diet by weight (Whitton et al., 2011). In most botanical sources of starch, the proportions of the glucan polymers, amylose and amylopectin, are typically in the range of ∼20−30 % and 70–80 %, respectively (Buléon, Colonna, Planchot, & Ball, 1998; Wang, Li, Copeland, Niu, & Wang, 2015). The supramolecular structure and properties of starch (*e.g.* gelatinisation and retrogradation) have an important bearing on digestion kinetics, which is known to impact on the extent and duration of postprandial glycaemia and insulinaemia (Dhital, Warren, Butterworth, Ellis, & Gidley, 2017; Edwards et al., 2020; Wang et al., 2015).

The first stage of starch digestion is amylolysis catalysed by α-amylase in saliva and then predominantly by amylase released from the pancreas. The resulting products of amylolysis (mainly maltose, maltotriose and maltodextrins) (Roberts & Whelan, 1960) are then hydrolysed to glucose by the dual function of disaccharidases, maltoglucoamylase and sucrase-isomaltase (Nichols et al., 2003). Glucose is then absorbed from the intestinal mucosa into the portal blood through the transporters GLUT2 and SGLT1 (Kellett & Brot-Laroche, 2005). Differences in the rate and extent to which starch is hydrolysed by α-amylase are important factors in explaining the large variations in postprandial glycaemia observed after ingestion of a starch-containing food. It is well known that the postprandial glycaemic response to plant foods with isoglucidic amounts of starch can differ greatly and responses are often described by the glycaemic index (GI) or glycaemic load (GL) values (Augustin et al., 2015; Edwards, Cochetel, Setterfield, Perez-Moral, & Warren, 2019; Jenkins et al., 2002). Low GI or GL diets are associated with a reduced risk of developing type 2 diabetes mellitus and cardiovascular disease (Augustin et al., 2015; Jenkins et al., 2002; Livesey et al., 2019). Fractions of starch that are not digested in the small intestine (*i.e.*, resistant starch or RS) are metabolised in the colon by microflora to short chain fatty acids such as acetate, propionate and butyrate and these metabolites are important for the maintenance of colonic epithelial cells and for affording protection against intestinal disease (Canani et al., 2011; Cummings, 1981; Topping & Clifton, 2001).

It is well known that hydrothermal processing of native, semi-crystalline starches substantially increases the susceptibility of α-glucan chains to amylase action due to a loss of ordered structure of the starch during gelatinisation (Baldwin et al., 2015; Dhital et al., 2017; Roder et al., 2009; Tahir, Ellis, Bogracheva, Meares-Taylor, & Butterworth, 2011). However, when gelatinised starch is cooled and stored, retrogradation of amylose and amylopectin occurs, where some of the α-glucan chains re-associate and become more structurally ordered, *e.g.* formation of amylose double helices (Gidley et al., 1995; Wang et al., 2015). Such double-helical α-glucan structures make α-(1→4) glucosidic linkages less accessible to α-amylase and are therefore more resistant to amylolysis (Gidley et al., 1995; Patel et al., 2017; Wang et al., 2015). Moreover, we recently demonstrated for the first time that retrograded starch is not only inert to amylolysis but also slows down the rate at which starch is hydrolysed by direct inhibition of pancreatic α-amylase (Patel et al., 2017).

Although numerous *in vitro* studies have shown that changes in the physico-chemical properties of starch induced by hydrothermal processing can greatly affect its digestibility (Baldwin et al., 2015; Dhital et al., 2017; Edwards, Warren, Milligan, Butterworth, & Ellis, 2014, 2015; Patel et al., 2017; Roder et al., 2009; Tahir et al., 2011), the effects of different heating, cooling and freezing treatments on digestion kinetics by amylase have not received a great deal of attention and findings are somewhat contradictory (Wang et al., 2015). Thus, in one report, the digestibility of starch in samples of hydrothermally-cooked beans (*Phaseolus vulgaris* L.) was found to be unaffected by storage at 4 °C for 96 h (Landa-Habana, Piña-Hernández, Agama-Acevedo, Tovar, & Bello-Pérez, 2004). In contrast, however, a subsequent study showed that hydrothermally-treated waxy maize starch (amylopectin rich), stored for 16 days under isothermal temperature (4 °C) conditions or 2-day cycled temperatures of 4 °C and then 30 °C, formed larger amounts of RS and reduced *in vitro* GI. (Park, Baik, & Lim, 2009).

There is an increasing consumer demand for pre-processed convenience food products and ‘ready-to-eat meals’ in which the ingredients are pre-cooked/processed into a meal that is subsequently refrigerated or frozen for retail, and then re-heated prior to consumption by the consumer. The starch contained within such products is likely to undergo structural transformations (*i.e.*, gelatinisation and retrogradation) at each processing stage, which will impact on digestion kinetics. However, the effects on the digestibility of the constituent starch subjected to freezing, storage and chilling regimes seem to have received scant attention, but the subject is a matter of considerable interest because of concerns over large postprandial glycaemic responses to certain food materials, especially in relation to increased risk of type 2 diabetes (Augustin et al., 2015; Livesey et al., 2019).

We now report the results of a structure-function study of the effects of different processing and storage treatments on *in vitro* digestibility of starch present in chickpea flour. The use of chickpeas as a source of starch for this mechanistic study was selected because of the considerable interest in the design of novel chickpea ingredients for foods with enhanced nutritional properties, notably with a high RS content and low glycaemic impact (Delamare et al., 2020; Edwards et al., 2020). After hydrothermal processing at 90 °C, the test samples were stored for various times with combinations of chilled (4 °C) or frozen (−70 °C) temperatures before estimation of the rates of starch digestion by pancreatic α-amylase. For the amylolysis assay, we made use of our recently developed Logarithm of Slope (LOS) analysis of experimentally-generated starch digestibility curves, to identify and quantify potential nutritionally important fractions (Edwards et al., 2014). This provided useful information on the rate processes that contribute to the amylolysis of pure starches and starches in complex food matrices. Solid-state ^13^C NMR was used to estimate the proportion of molecular order, specifically the ordered double-helical α-glucan structure of starch in the legume samples, to aid interpretation of the digestibility data.

## 2 Materials and methods

### 2.1 Chickpea flour

Whole chickpeas (*Cicer arietinum* L., Russian cv., Kabuli type) were supplied by AGT Poortman (A. Poortman (London) Ltd., UK) and dry-milled into flour at the VTT Technical Research Centre of Finland Ltd. Whole chickpeas were first crushed with a cutting mill (Retsch SM300, Germany) using a 4 × 4 mm sieve and 700 rpm speed and then ground in a 100UPZ pin disc mill (Hosokawa Alpine, Germany) at 17800 rpm. This resulted in a flour with a unimodal particle size distribution (percentage volume size) with parameters d10, d50, d90 = 7, 19 and 55 μm, respectively, and diameters of particles assumed to be spherical (see **OSM 1**). The particle size analysis was performed in duplicate using a Beckman Coulter LS 230 (Beckman Coulter Inc, CA, USA) with ethanol as a carrier.

Proximate composition of the chickpea flour was determined by UKAS accredited laboratory, ALS Food and Pharmaceutical Ltd., Chatteris, UK. Protein was determined by Dumas nitrogen using a conversion factor of 6.25, total lipid was by determined by NMR (using a 0.956 conversion factor for non-fatty acid material in the lipid), fatty acid composition was analysed by GC using flame ionisation detection and ash (total minerals) was determined by combustion in a furnace. Total dietary fibre was determined by AOAC Official Method 991.43 (a gravimetric and enzymic method), and total sugars content was determined by ion-exchange HPLC. The ‘available’ carbohydrate content was calculated ‘by difference’. Energy values were calculated using standard conversion factors. In addition, direct analyses of total starch and moisture content of chickpea flour at the time of the digestibility experiments were performed in-house to provide a more accurate measure of starch content than the ‘by difference’ value. The moisture content of quadruplicate samples was determined gravimetrically by drying overnight in a forced-air (fan) oven (Gallenkamp Hotbox) at 103 ± 2 °C. The starch content was determined using the DMSO protocol of the Megazyme Total Starch Method, AOAC 996.11, (Megazyme International, Bray, County Wicklow, Ireland) with some modifications (Edwards et al., 2014; Edwards et al., 2015).

### 2.2 Reagents for amylolysis assay

Porcine pancreatic α-amylase (PPA, EC 3.2.1.1) of high purity (Grade 1-A) was obtained from Sigma-Aldrich Co. Ltd, Poole, Dorset, UK (A6255). The enzyme was supplied as a suspension in 2.9 mol/L NaCl containing 3 mmol/L CaCl_2_. Quality control tests of the PPA described in our previous paper (Edwards et al., 2014) showed that the enzyme was highly pure and the total protein and enzyme activity were within the range specified by the manufacturer. One unit of activity, as defined by the manufacturers, releases 1 mg of maltose from starch in 3 min at 20 °C. This is approximately equivalent to 1 IU/mg protein at 20 °C. Phosphate buffered saline (PBS), pH 7.3 ± 0.2, was prepared from tablets following the manufacturer’s instructions (Oxoid Ltd., Basingstoke, Hampshire, UK).

### 2.3 Processing and storage regimes used for chickpea flour preparations

An overview of the processing treatments and corresponding sample codes is provided in Table 1. Each letter in the sample code reflects the order and type of treatment applied to the chickpea flour samples. These treatments were designed to simulate conditions applied to legume products processed commercially and domestically. The codes N and G are used for defining native and gelatinised, respectively, and refer to the physical state of the starch in the chickpea flour samples.

**Table 1:** Overview of processing treatments used for manipulating starch digestibility in chickpea flours.

Code	Treatment	Description of processing and storage regimes
N	Native	Flour suspended in PBS (7.5 mg flour/mL), with continuous stirring
G	Gelatinised	‘N’ treated in a water bath at 90 °C for 20 min, with continuous stirring
O	Oven-dried	Dried in a forced air oven at 100 °C for 24 h^a^
I	Incubator-dried	Dried in an incubator at 40 °C for 48 h^a^
A	Air-dried	Dried at ambient temperature (∼22 °C) for 72 h^a^
F	Refrigerated	Refrigerated and stored at 4 °C for 72 h
Z	Frozen	Frozen and stored at −70 °C for 60 h^b^

Treatments (coded O, I, A, F and Z) were applied to native (N) and/or gelatinised (G) samples. ^a^PBS was added to dried samples to reconstitute to the original concentration prior to use. ^b^Frozen samples were thawed at room temperature for 16 h prior to use. N.B., the differences in drying time between oven-dried, incubator-dried and air-dried materials were linked to the temperature differences and therefore rate of drying. Dried flour samples of similar appearance were produced (*i.e.* no differences in colour and texture were observed).

All samples were prepared from a starting solution of either native (raw) or gelatinised (heated at 90 °C) starches in stock preparations of chickpea flour suspended in PBS (7.5 mg flour/mL PBS), with a starch concentration of 4 mg/mL. For native chickpea flour stock, the raw chickpea flour was weighed directly into a flask and combined with PBS (prepared at room temperature) and then stirred to form a suspension. The chickpea flour suspensions with native starch were either analysed as is (denoted N) or subjected to further processing steps as listed in Table 1 (samples with treatment code N as the first letter).

For gelatinisation of the starch in the chickpea flour, the PBS was heated to 90 °C with constant stirring on a magnetic stirrer with a temperature sensor (RET basic, IKA®) and then the flour was sprinkled into the vortex of the solution and stirring continued for a further 20 min at the same high temperature and then allowed to cool for 10 min at room temperature (∼22 °C) before further processing. All samples treated in this way were labelled with the treatment code ‘G’. For further processing and storage of the native or gelatinised samples, 4 mL aliquots of the stock suspension were transferred to 15 mL Falcon tubes or to aluminium pans (dependent on further treatment) and then processed in the following ways before use in digestibility assays with PPA:

i) *Drying treatments:* Native (N) and gelatinised (G) samples were transferred into aluminium pans and heat-processed in either a forced air (fan) oven (Gallenkamp Hotbox), at approximately 100 °C for 24 h (samples NO and GO), or in an incubator (LEEC Compact Incubator, LEEC Ltd., Nottingham, UK) at approximately 40 °C for 48 h (samples NI and GI), or left to air dry (samples NA and GA) on a laboratory bench under ambient conditions, ∼22 °C for 72 h. After drying, PBS was added and vortex mixing applied to the dried samples to reconstitute these to the standardised starch concentration (4 mg/mL) required for the amylolysis assay.
ii) *Cold treatments:* The cooled gelatinised (G) samples were transferred into 15 mL Falcon tubes and either immediately refrigerated at 4 °C for 72 h (treatment code F), or frozen at −70 °C and stored at this temperature for 60 h, and then defrosted for 16 h at room temperature (treatment code Z). Samples GF and GZ were then brought to 37 °C and analysed immediately.
iii) *Treatment Combinations*: To assess the effect of reheating cold-stored samples on starch amylolysis, the cold-stored samples denoted GFG and GZG were subjected to the gelatinisation treatment (G) once more after refrigeration or freezer storage (treatments F and Z). These treatments were also applied in combination to examine the effect of including a refrigeration step before (sample GFZG) or after (sample GZFG) the freezing treatment and prior to repeating the gelatinisation treatment.

All samples were prepared fresh for analysis and equilibrated to 37 °C prior to amylolysis assay.

### 2.4 *In vitro* digestibility measurements and analysis of digestibility profiles

Following various processing treatments (see Section 2.3), the flour sample suspensions (containing 4 mg/mL starch) were incubated at 37 °C. Full details of the *in vitro* digestion method are given in a previous publication (Edwards et al., 2014). In brief, the digestion assay was initiated by the addition of PPA to provide a final enzyme concentration of 8 nM in a reaction mixture of 10 mL. Digestion was continued for up to 90 min in tubes subjected to constant end-over-end mixing with withdrawal of 100 μL samples at appropriate time intervals. The samples were then added to 150 μL of stop solution consisting of ice-cold 0.3 M Na_2_CO_3_ and immediately centrifuged for 5 min at 16,200 × *g* to sediment any undigested material. Aliquots (150 μL) of the supernatant were then transferred to a 96-well microplate for determination of reducing sugars by the Prussian blue assay (Edwards et al., 2014) and expressed as maltose equivalents after reference to a standard curve performed with maltose (Slaughter, Ellis, & Butterworth, 2001). Four replicates of the digestibility assay on each processed sample was performed.

The digestibility curves of the starch-containing chickpea flours were subjected to Logarithm of Slope (LOS) analysis of pseudo-first-order kinetics to determine rate constants *k*, and *C*_∞_*,* the total amount of digestible starch, the full details of which are published elsewhere (Butterworth, Warren, Grassby, Patel, & Ellis, 2012; Edwards et al., 2014). The values of *k* and *C*_∞_ are estimated from the slope (*k*) and y-intercept (ln [*C*_∞_*k*]), respectively, of the linear plots of LOS *versus* time of amylolysis. The LOS plot method was previously validated for use in the analysis of first-order kinetic data, not just on pure starches but also starch-containing edible plant tissues (Edwards et al., 2014), including the processed chickpea flours selected for the current study. In food ingredients and products containing starch fractions that are digested at different rates, the LOS plots can produce two or more distinct linear phases. These linear plots show single or two phases of amylolysis and allow calculation of digestibility rate constants (*k*_1_ and *k*_2_) and end-point starch amylolysis (*C*_1∞_ and *C*_2∞_) for phases 1 and 2, respectively. In addition, a C90 value, which is the percentage of hydrolysable starch digested at 90 min, was estimated from the first-order kinetic model described previously (Edwards et al., 2014) and is a useful *in vitro* predictor of the GI of foods (Edwards et al., 2019).

### 2.5 NMR method

NMR analysis (Flanagan, Gidley, & Warren, 2015) was performed on a sub-set of the processed chickpea flour samples (N, G, GF and GZ) before and after the amylolysis assay. Flour samples collected before digestion were processed (as described above) and freeze-dried immediately. Digested samples collected after 90 min amylolysis were centrifuged (16,200 × g for 6 min; Haraeus Pico, Thermo Scientific) to exclude the supernatant (containing the starch digestion products of mainly maltose), and the resulting pellets, containing the RS that remained after digestion, were freeze dried and powdered using a pestle and mortar before NMR spectra were recorded.

^13^C Cross Polarisation – Magic Angle Spinning (CP-MAS) NMR was performed at 100.61 MHz for ^13^C and 400.13 MHz for ^1^H on a Bruker Avance 400. The samples were placed in 4 mm, partially filled rotors and spun at a MAS frequency of 13 KHz with the temperature maintained at 298 K. The contact time was 2 ms and acquisition time was 50 ms. Spectra were externally referenced to adamantane (28.46 and 37.85 ppm). Calculation of molecular order (double-helical content) of the starch was achieved using the method of Flanagan et al. for analysis of the spectra (Flanagan et al., 2015). Examples of NMR spectra of starch samples can be found online (**OSM 2**).

### 2.6 Statistical analysis

All data are presented as means ± SEM (4 replicates) unless otherwise specified. The values of *k* and *C*_∞_ were estimated from the slope and y-intercept, respectively, of the linear plots of LOS *versus* time of amylolysis using regression analysis. One-way analysis of variance (ANOVA) was performed on the C90 digestibility data. Statistically significant differences were accepted at the *P* < 0.05 level. The analysis was performed using IBM SPSS Statistics 20.0. All other analyses were performed using SIGMAPLOT 12.0 (©Systat software 2011) statistical and graphical software.

## 3 Results

### 3.1 Proximate analysis of chickpea flour

The chickpea flour contained (per 100 g, as is) 18.6 g protein, 5.2 g lipid, 3.0 g ash, 12.1 g total dietary fibre, 52.5 g available carbohydrate (calculated by difference) of which 3.4 g was total sugars and the calculated total energy value was 1500 kJ per 100 g. The lipid content comprised 0.74 g saturated, 1.23 g monounsaturated and 2.99 g polyunsaturated fatty acids. Direct analysis of the starch content in the chickpea flour was found to be 53 ± 2 % (dry weight basis), and the moisture content of the original flour was 9.7 ± 0.3 %.

### 3.2 Digestibility curves of starch in chickpea flour samples processed and stored under different regimes

**Fig. 1:**
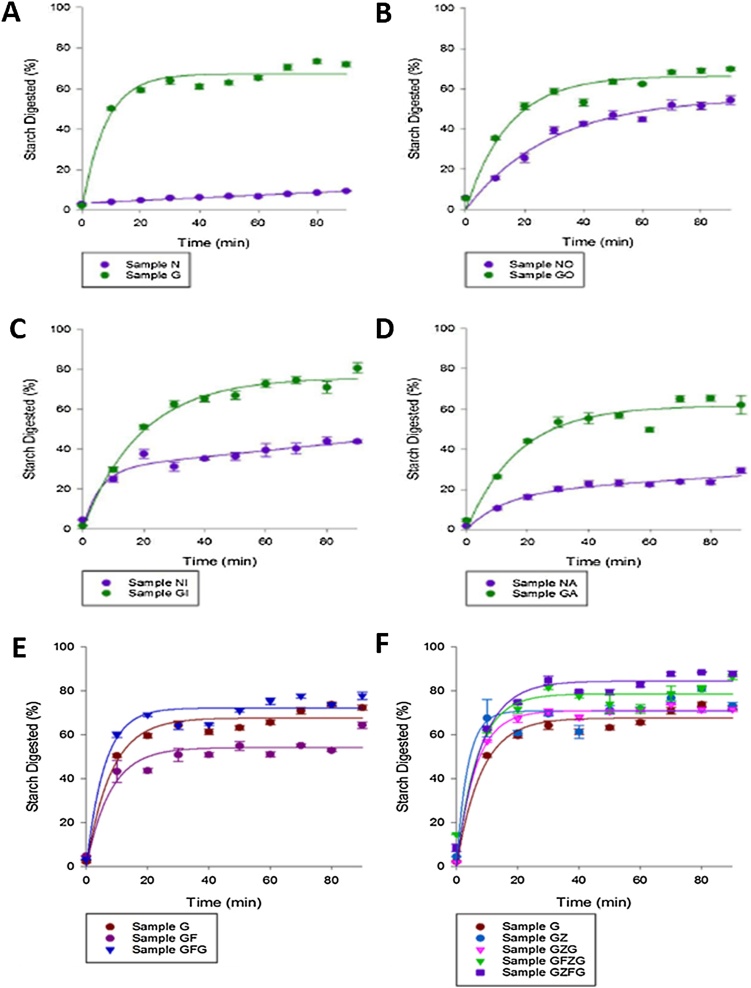
Starch digestibility curves of native and gelatinised starch in chickpea flour samples following different processing and storage regimes. All values are means ± SEM (n = 4). A, Control; B, Processed by oven treatment; C, Processed by incubator treatment; D, Processed by air drying; E, Processed by refrigeration; F, Processed by freezing. The sample code in each legend denotes the processing treatment: Native starch, ‘N’; Gelatinised starch, ‘G’; Oven-dried, ‘O’; Incubator, ‘I’; Air-dried, ‘A’; Refrigerated, ‘F’, and Frozen, ‘Z’. These letters are in the order that each treatment was applied to the sample. For full details of processing treatments refer to Section 2.3 and Table 1.

Typical digestibility curves obtained for native and gelatinised starch in the chickpea flour samples subjected to various processing regimes and storage conditions are shown in Fig. 1. Native starch (N) was most resistant to amylolysis and the gelatinisation treatment (G) led to a major increase in the rate and extent of amylolysis (Fig. 1A). Treatment of native samples in an oven, incubator or by air-drying was also associated with an increase in the susceptibility of starch to amylolysis. However, the oven and incubator drying treatments of gelatinised materials (GO and GI) had less clear effects on starch digestibility, although the early digestion phase (0−20 min) was attenuated for these dried samples (Fig. 1B,C) compared with gelatinised starch (G in Fig. 1A). In the case of the air-dried sample (GA), this produced a lower rate and extent of amyloysis over the whole 90 min digestibility period relative to the gelatinised sample (Fig. 1A,D). Refrigeration of the gelatinised sample (sample GF) lowered its starch digestibility; but notably, when the gelatinisation treatment was re-applied to this sample after cold storage (sample GFG) the starch became more digestible than the original gelatinised sample, G (in Fig. 1E, amylolysis for samples GF < G < GFG). As seen in Fig. 1F, the gelatinised samples that were frozen (without a refrigeration step) had a similar starch digestibility to the gelatinised sample (amylolysis for samples G ∼ GZ and GZG). However, when refrigeration (F) was used in combination with freezing (Z) and the gelatinisation treatment repeated, the starch became more digestible than the original gelatinised sample (in Fig. 1F, amylolysis for samples GFZG and GZFG > G, and for samples G ∼ GZG ∼ GZ). Thus, Fig. 1F shows that freezer storage following gelatinisation had negligible effect on starch digestion even after repeated hydrothermal processing, but when coupled with refrigeration the starch appears to be rendered more susceptible to amylolysis.

**Fig. 2:**
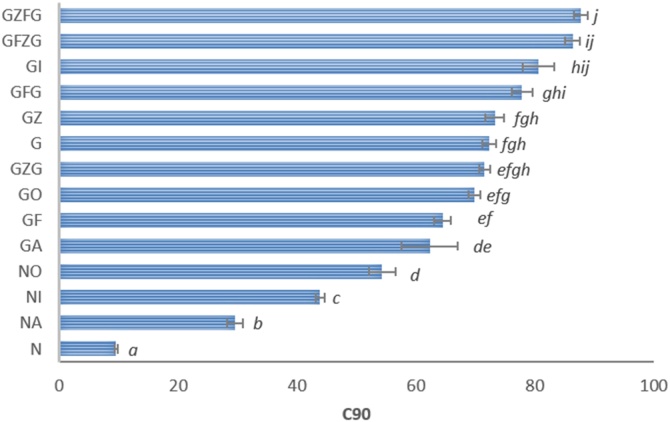
Effect of processing and storage treatments on the extent of starch digestion (%) after 90 min (C90). All values are means ± SEM (n = 4). The sample code denotes the processing treatment: Native starch, ‘N’; Gelatinised starch, ‘G’; Oven-dried, ‘O’; Incubator, ‘I’; Air-dried, ‘A’; Refrigerated, ‘F’; and Frozen, ‘Z’, and these letters are in the order that each treatment was applied to the sample. For full details of processing treatments refer to Section 2.3 and Table 1. C90 mean values labelled with the same letter are not significantly different (one-way ANOVA, P ≥ 0.05).

The large differences in amylolysis are illustrated in Fig. 2, which shows the extent of starch digested after 90 min exposure to amylase and provides an indication of likely relative differences in the potential glycaemic responses to starch-rich foods *in vivo* (Edwards et al., 2019). The differences in C90 values between samples are compatible with the digestibility profiles seen in Fig. 1. Thus, as expected, the C90 values of the native starches, including the ones dried by different methods, were much lower than all the gelatinised starch materials, of which the highest values (> 80 %) were found for samples that had received a second hydrothermal treatment subsequent to frozen and chilled (refrigerated) storage; *i.e.*, GZFG and GFZG. It is also worth noting the statistically significant lower C90 value for the gelatinised air-dried (GA) sample relative to freshly gelatinised starch (G).

### 3.3 LOS plot analysis of digestibility curves and calculation of kinetic parameters k and C_∞_ of starch in chickpea flour samples

**Fig. 3:**
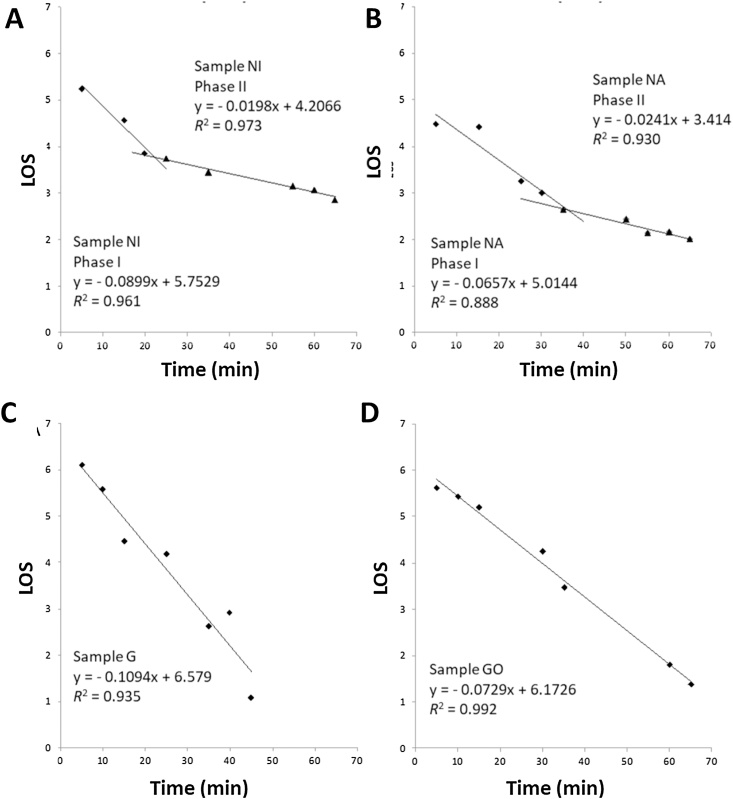
LOS plots of digestibility data obtained for native and gelatinised starch in chickpea flour samples subjected to different processing regimes. The plots show single or two phases of amylolysis, as explained in Methods Section 2.4; regression analysis of the linear plots allow calculation of values of *k* and *C_∞_* for phases 1 and 2, as seen in Table 2. Examples of LOS plots obtained from: A, Native starch sample dried in an incubator (NI); B, Native starch sample that was air-dried (NA); C, Gelatinised starch sample that received no processing (G, control); and D, Gelatinised starch sample that was oven heated (GO). LOS plots for the other chickpea samples are included in the online supplementary information (**OSM 3**).

Digestibility curves of the type shown in Fig. 1 were then analysed using LOS plots (for examples see Fig. 3) to obtain values for digestibility constants *k*, the rate constant, and *C_∞_*, the total amount of starch in chickpea flours that can be digested. Native (*i.e.*, non-gelatinised) starch in the chickpea samples that had been incubator- or air-dried generated biphasic LOS plots from which *k* and *C_∞_* values for each stage were calculated. The resulting kinetic parameters obtained for samples following different processing regimes are summarised in Table 2. As expected, the chickpea flours containing gelatinised starch were found to have a vastly greater *in vitro* digestibility (*C*_∞_) than the native samples, irrespective of the processing treatment. The exceptions to this were the air-dried gelatinised sample (GA), which had a *C*_∞_ value similar to dried native samples (with a range of values between 30.2–58.5 %; Table 2), and the refrigerated (GF) sample that had a slightly lower *C*_∞_ than gelatinised alone (G). The digestibility constant, *k*, for gelatinised samples and for those that had been re-treated by hydrothermal processing after refrigeration and freezing, were essentially identical (mean value of 0.112 min^−1^ with S.D. of 0.014) (Table 2). With the exception of the native sample that had been treated in an oven at 100 °C for 24 h (*i.e.*, NO), the digestibility constants for phase 1 (*k_1_*) of native samples closely matched the values for the gelatinised samples. For freely available substrates *k* values are expected to be virtually identical since this an inherent property of amylase under these assay conditions.

**Table 2:** *C*_∞_ and *k* parameters estimated from LOS analysis of starch in chickpea flour samples digested under different processing and storage conditions.

	Single or first phase		Second phase		Total *C*_∞_ (%)
	*C*_1∞_ (%)	*k*_1_ (min^−1^)	*C*_2∞_ (%)	*k*_2_ (min^−1^)	
(N)	–	–	–	–	(C90 = 9.8)^a^
(NO)	50.1	0.03	–	–	50.1
(NI)	36.0	0.09	22.5	0.02	58.5^b^
(NA)	23.5	0.07	6.7	0.02	30.2^b^
(G)	67.6	0.11	–	–	67.6
(GF)	64.5	0.05	–	–	64.5
(GFG)	84.0	0.07	–	–	84.0
(GZ)	75.9	0.05	–	–	75.9
(GZG)	73.4	0.15	–	–	73.4
(GFZG)	70.5	0.15	–	–	70.5
(GZFG)	86.1	0.08	–	–	86.1
(GO)	67.5	0.07	–	–	67.5
(GI)	79.6	0.06	–	–	79.6
(GA)	55.9	0.08	–	–	55.9

*C*_∞_ represents the total starch available for digestion and *k* is the digestibility constant. These parameters were obtained by LOS plot analysis of digestibility curves with single (first) or second phases and are mean values of 4 replicates. For details see Methods section 2.4. Each letter in the sample code reflects the processing treatment applied (N, Native; G, Gelatinised; O, Oven-dried; I, Incubator-dried; A, Air-dried; F, Refrigerated; Z, Frozen; see Section 2.3 and Table 1) and the order of the letters reflect the treatment order.

a *C*_∞_ could not be determined for native starch (N) and so the percentage of starch digested at the 90 min is shown.

b Reactions that were biphasic and the total *C*_∞_ is a summation of values obtained for each phase.

### 3.4 NMR analysis of starch in chickpea flour samples processed and stored under different regimes

Data from solid-state ^13^C CP-MAS NMR were used to estimate the proportion of molecular order, specifically the ordered double-helical structure of the starch in the chickpea flour samples (Table 3). The amount of double-helical structure in the native sample (N) was 25 % and after digestion with amylase it was 24 %, so that this quantity was hardly changed by digestion with amylase. The total amount of starch digested in N (*C*_∞_, expressed as a dry weight basis, dwb) was only 10.7 %. Since the amount digested after 90 min of incubation was less than 11 % of the total starch, it is hardly surprising that there was minimal change in the detectable double-helical content. The double-helical content (ordered structure) decreased to <5 % after starch gelatinisation (sample G) and this had a total amylase digestibility of 74.1 % (dwb). After refrigeration of the gelatinised starch (GF) for 72 h at 4 °C, the helical content increased to 10 % and *C*_∞_ was 70.8 % (dwb). Following digestion with amylase the content of ordered material in GF was increased further to 13 %. No increase in double-helical structure was observed for the gelatinised-frozen starch sample (GF) and this remained at <5 %. The *C*_∞_ value for amylase digestibility of GF was 83.2 % (dwb). After amylase digestion of GF, the percentage of double-helical order increased to 14 %.

**Table 3:** Double-helical (DH) order and digestibility of starch in chickpea flour samples^a^ following different processing/storage treatments; DH calculated before and after digestion with pancreatic α-amylase.

Treatment		Before digestion (% DH order)	After digestion (% DH order)	*C*_∞_ (% dwb)
Native	(N)	25	24	(C90 = 10.7)^b^
Gelatinised	(G)	<5	<5	74.1
Gelatinised and refrigerated	(GF)	10	13	70.8
Gelatinised and frozen	(GZ)	<5	14	83.2

a Samples were freeze-dried prior to NMR spectroscopy and the *C*_∞_ values are presented on a dry-weight basis (dwb).

b The value for native starch (N) is the percentage of starch digested with α-amylase at the 90 min incubation time point because the digestibility rate was very slow and the curve was not suitable for LOS analysis.

## 4 Discussion

Proximate compositional analysis of the chickpea flour showed that it was rich in starch, protein and dietary fibre at levels consistent with values reported previously (Wood & Grusak, 2007). We have subjected the starch contained in chickpea flour to combinations of hydrothermal processing (to gelatinise the starch), plus various drying methods, and conditions of freezer and chilled storage. These regimes were selected to simulate the kinds of processing and storage conditions that prepared ready meals/foods may encounter, so that the impact on starch structure in relation to its susceptibility to amylolysis could be investigated. The order and combination in which these treatments were applied to the same flour preparation were found to have a major impact on starch digestibility. Our results from the digestibility profiles, and the LOS plot and solid-state ^13^C NMR analyses suggest that the marked differences in the rate and extent of amylolysis resulting from the various storage treatments can be attributed to changes in the ordered structure of starch and the number of free α-glucan chains available to amylase.

It is important to point out that the explanation for the well-documented variations in the rates of amylolysis in different food matrices is not completely understood. However, there are many food-related factors, in addition to the processing and storage conditions mentioned above, that are known to contribute to the differences in starch digestion kinetics (Dhital et al., 2017). These include the structural characteristics and properties of the starch and food matrix (*e.g.* degree of gelatinisation and intactness of cell walls in edible plants) and the presence of protein, lipid and phenolic compounds (Edwards et al., 2014; Edwards et al., 2015; Slaughter et al., 2001; Tahir et al., 2011). In the present study, we have controlled for potential confounding variables, such as variations in composition and physical characteristics, by testing the same leguminous flour preparation only. Thus, any differences in amylolysis can be mainly attributed to variations in processing and storage conditions.

As expected, native starch in raw chickpea flour was the most resistant to digestion (∼10 % starch digested after 90 min) and had a high proportion of double-helical order (∼25 %). When hydrothermal processing (for 20 min at 90 °C) was applied to chickpea flour to gelatinise the starch, the double-helical order was lost, which increased the availability of disordered α-glucan chains and thereby greatly increased the susceptibility of the gelatinised sample to amylolysis. These effects are consistent with a plethora of literature reporting substantial increases in the rate and extent of starch digestion following hydrothermal processing due to the loss of ordered structure and an increase in available starch (Dhital et al., 2017; Roder et al., 2009; Tahir et al., 2011). In a mechanistic *in vitro* study of amylolysis, using solution-state NMR combined with enzyme kinetics, we demonstrated that an increase in the number of flexible, highly mobile α-glucan chains, protruding from the exposed surface of starch granules during gelatinisation, are the primary substrate for pancreatic α-amylase (Baldwin et al., 2015; Dhital et al., 2017).

Although the amylolytic process of native and gelatinised starches is reasonably well understood (Baldwin et al., 2015; Dhital et al., 2017; Edwards et al., 2014, 2019), we still have limited insight of the physico-chemical properties of retrograded starch and indeed other forms of RS when present in food materials during digestion and their impact on postprandial glycaemia (Patel et al., 2017). Moreover, the effects of heat processing, including re-heating, and storage regimes on the behaviour of retrograded starch during amylolysis are not well understood and results of studies in this area are inconsistent (Wang et al., 2015). In the present study, refrigeration of samples (at 4 °C for 72 h) post-gelatinisation resulted in starch retrogradation and impaired digestibility by amylase, whereas gelatinised samples that were immediately frozen (at −70 °C and stored for 60 h) were not significantly different from the original gelatinised sample after 90 min starch digestion.

Evidence that the decrease in digestion is attributed to an increase in starch retrogradation when gelatinised starch is refrigerated, is derived from the solid-state ^13^C NMR data, which revealed that the proportion of double-helical order increased from <5 % for the gelatinised starch to 10 % when the same sample was stored at 4 °C. The effect of this storage in generating slowly-digestible starch in the chickpea flour probably arises from re-association of α-glucan chains during retrogradation. In our previous study, employing LOS plot analysis of digestibility profiles, starches containing retrograded material showed unchanged digestibility rate constant *k*, but a decrease in *C*_∞_ values (total digestible starch) relative to freshly-prepared gelatinised starches (Patel, Day, Butterworth, & Ellis, 2014). These results suggested that retrograded starch is virtually inert to α-amylase action over the time course of the experiment (Patel et al., 2014). In a later study, we reported that a purified sample of retrograded starch, prepared from high amylose maize, slowed down the rate of starch digestion by direct inhibition of α-amylase (Patel et al., 2017). The current experiments were not designed in the same way and used starch-containing leguminous flours, but it is of interest to note that the refrigerated starch sample possessed a lower *C*_∞_ value and digestibility profile than the freshly cooked sample. Furthermore, when these materials were analysed by NMR before and after amylolysis the proportion of double-helical order in the starch was higher after digestion. It is possible that some retrogradation may have occurred in all samples during freeze-drying and perhaps even during digestion itself, as previously reported, albeit in high-amylose maize starches (Htoon et al., 2009). Htoon and colleagues suggested that increases in order/crystallinity are likely during enzyme digestion in these types of starch. The marked difference in crystallinity observed for the gelatinised-refrigerated sample suggests that amylase action is mainly focussed on the non-ordered regions of starch with flexible glucan chains, such that starch remaining after the digestion had a higher proportion of double-helical order (Baldwin et al., 2015; Dhital et al., 2017).

Interestingly, the digestibility profiles showed that any impaired digestibility as a consequence of storage of the gelatinised sample at 4 °C (sample GF) was reversed by re-heating (sample GFG) and indeed became more digestible than the original gelatinised sample (G). This increase in susceptibility to amylolysis after re-heating refrigerated samples suggests that the re-associated α-glucan chains in the retrograded starch were destabilised by hydrothermal processing. This result is consistent with a recent study showing that starch potato paste, refrigerated for 2 days at 4 °C, was more susceptible to digestion after microwave re-heating compared with freshly made hydrothermally cooked starch (Colussi et al., 2017). Additionally, it seems that the method we used in our initial hydrothermal processing to bring about gelatinisation did not result in a complete disruption of the ordered structure. Thus, a contributing mechanism may be that the second hydrothermal processing cycle increased the number of flexible glucan chains potentially available for hydrolysis by α-amylase. The relatively high *k* values (0.15 min^−1^, comparable to gelatinised starch) seen after the repeated hydrothermal processing of frozen samples also provides evidence of further loss of ordered structure.

The rate of amylolysis and total amount of starch available for digestion after gelatinisation was considerably decreased by air drying (held at 22 °C for 72 h) and it is likely that this drying method potentiated the retrogradation of starch, particularly the amylose fraction (Haralampu, 2000; Htoon et al., 2009; Patel et al., 2014). However, the type of drying method appears to be important because the other drying methods (oven and incubator) had little or no effect on amylolysis, compared with non-dried samples, except for a decrease in the first 20 min digestion period. Indeed, the *C*_∞_ and C90 values for the oven and incubator-dried samples showed similar or increased levels of digestion relative to freshly gelatinised material. The biphasic LOS plots obtained for native and air-dried fractions suggest heterogeneity within these samples, with the fraction digested during phase 1 assumed to be readily available surface starch that has exposed flexible polyglucan chains (Baldwin et al., 2015; Edwards et al., 2014). These mobile disordered glucan chains are the primary substrates for α-amylase action and are easily removed in early stages of amylolysis (Baldwin et al., 2015). Thus, such flexible chains are likely to be digested at rates commensurate with that seen for gelatinised starch, which has a proportionately greater number of these disordered chains and represents the intrinsic reactivity of α-amylase on amorphous starch. The lower *k* value for starch granules subjected to air drying may result from a loss of some flexible surface polyglucan chains that occurred during drying, leading to attenuated amylolysis. These findings are consistent with data from a previous study, in which after 90 h of storage of gelatinised pea starch at ambient temperature, there was a decrease in catalytic efficiency, brought on by α-amylase binding to retrograded starch (Patel et al., 2017). In the present study, the rate of moisture loss from the starch-rich samples dried under different conditions is also likely to be important (Roder et al., 2009), but it is not clear if the differences between drying treatments are due to the heat-moisture conditions, the storage time period or a combination of these factors.

The C90 values are particularly useful in providing some indication of the relative differences in glycaemic responses that could be anticipated *in vivo* (Edwards et al., 2019), and suggest that the gelatinised samples treated by air-drying could potentially attenuate postprandial glycaemia compared with freshly gelatinised starch. In the case of the chilled (4 °C) starch sample (GF), as discussed above, the data provided some evidence of retrogradation and lower levels of digestion, although the decreased C90 value was not statistically significant, suggesting that any reduction in GI would be minimal. Notably the C90 value for frozen storage of cooked starch was virtually identical to that obtained for the freshly gelatinised starch, which is consistent with the other digestibility parameters (*e.g.* C∞) mentioned above. Furthermore, samples of gelatinised starch that were refrigerated and frozen, irrespective of the order of this cooling/storage treatment, showed a significant increase in starch digestibility following re-heating. With the growing popularity of consumption of convenience foods that are sold by supermarkets in pre-frozen or chilled states, it is important that the digestibility properties of these foods are understood to enable sound dietary advice to be available for consumers. The widely reported increase in levels of obesity, especially in children, is of great concern because of the links of obesity with raised risk factors for cardiovascular disease and type 2 diabetes (Augustin et al., 2015; Jenkins et al., 2002; Livesey et al., 2019). If this behaviour of the chickpea flour is mirrored by the starch found in whole foods, frozen-prepared meals composed of highly digestible forms of starch are likely to produce large peaks in postprandial glycaemia and insulinaemia and thus continue to be designated as foods with unfavourably high glycaemic indices (Edwards et al., 2014, 2015, 2019).

## 5 Conclusions

Overall, the results of our study have demonstrated marked differences in amylolysis between samples of starch-containing chickpea flour that has been processed and stored under regimes that were selected to simulate conditions occurring during the commercial preparation of ready meals. Hydrothermal processing treatments that gelatinise starch have a profound impact on starch susceptibility to amylase action, and although the rate and extent of amylolysis may be attenuated somewhat by the formation of retrograded starch during refrigeration treatment, these effects were found to be reversed during a second cycle of hydrothermal processing. The air-drying treatment applied to gelatinised starch was found to be particularly effective in lowering the susceptibility of starch to amylolysis. Further work is required to improve mechanistic understanding of the structural changes in starch that occur during processing and storage. This is likely to inform food processing strategies designed to promote the formation or preservation of RS in food products for applications in digestive and cardiometabolic health.

## Author contributions

CE, PB, PE, and JM designed and supervised the experiments; AV performed the experiments; AV, JM and CE analysed the data; CE, PB and PE wrote the paper and JM and AV contributed to subsequent versions of the manuscript. PE had final responsibility for the manuscript content. All authors read and approved the final version of the manuscript.
