# Supplementary material for: α-Amylase action on starch in chickpea flour following hydrothermal processing and different drying, cooling and storage conditions
Source: Carbohydr Polym. 2021 May 1;259:117738. doi: 10.1016/j.carbpol.2021.117738 (PMC7965859; doi:10.1016/j.carbpol.2021.117738)
Supplement: Supplementary file 1 [file mmc1.docx]

**Title:** α-Amylase action on starch in chickpea flour following hydrothermal processing and different drying, cooling and storage conditions

**Authors:** Edwards, C.H., Veerabahua, A.S., Mason, A.J., Butterworth, P.J., Ellis, P.R.

**Online Supplementary Material (OSM)**

**OSM 1.** Micrograph of raw (untreated) chickpea flour examined by light microscopy showing free starch granules stained with Lugol’s Iodine (aqueous solution of potassium iodide and iodine) and milled particles of chickpea tissue.


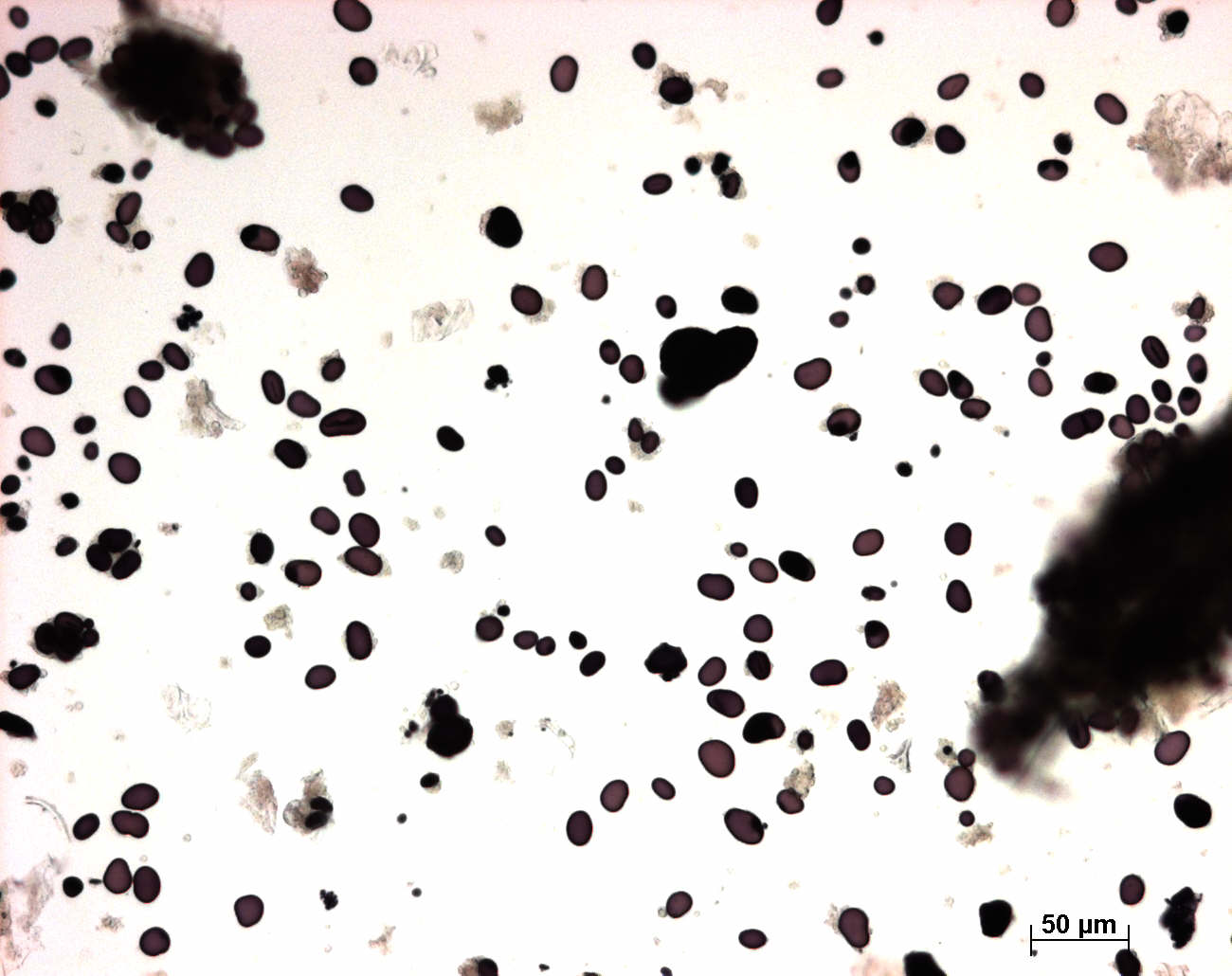


**OSM 2.** Normalised 13C CP-MAS NMR spectra for gelatinised (black) and native (red) starch in chickpea flour before digestion with amylase. Peaks are assigned to each carbon atom in the glucose ring.


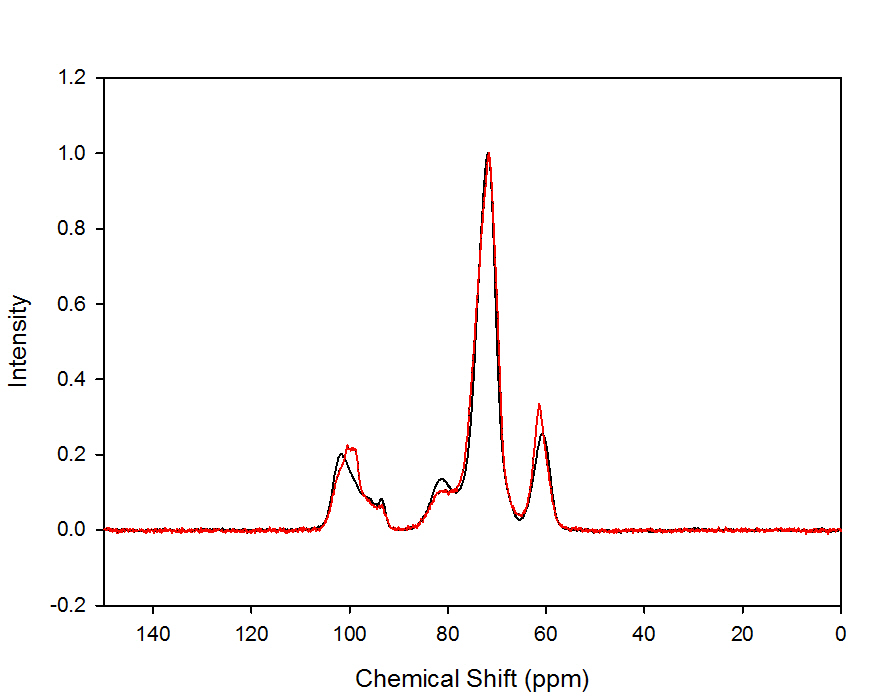


C1 C4 C2,3,5 C6

**OSM 3.** Additional LOS plots of digestibility data obtained for starches in chickpea flour samples subjected to different processing, drying and storage regimes (plots for native and gelatinised and dried samples can be seen in Fig. 3 of the manuscript). The plots show single phases of amylolysis, as explained in Methods section 2.4; regression analysis of the linear plots allow calculation of values of *k* and *C*∞ for phases 1 and 2, as seen in Table 2. Sample types: A, Gelatinised air-dried (GA) sample; B, Gelatinised incubator (GI) sample;
C, Gelatinised refrigerated (GF) sample; D, Gelatinised refrigerated and re-gelatinisation treatment (GFG) sample; E, Gelatinised frozen (GZ) sample; F, Gelatinised frozen and re-gelatinisation treatment (GZG) sample; G, Gelatinised refrigerated, frozen and re-gelatinisation treatment (GFZG) sample; H, Gelatinised frozen, refrigerated and re-gelatinisation (GZFG) sample; and I, Native oven (NO) sample. These letters are in the order that each treatment was applied to the sample. For full details of processing treatments refer to the main manuscript (Section 2.3 and Table 1).

**_
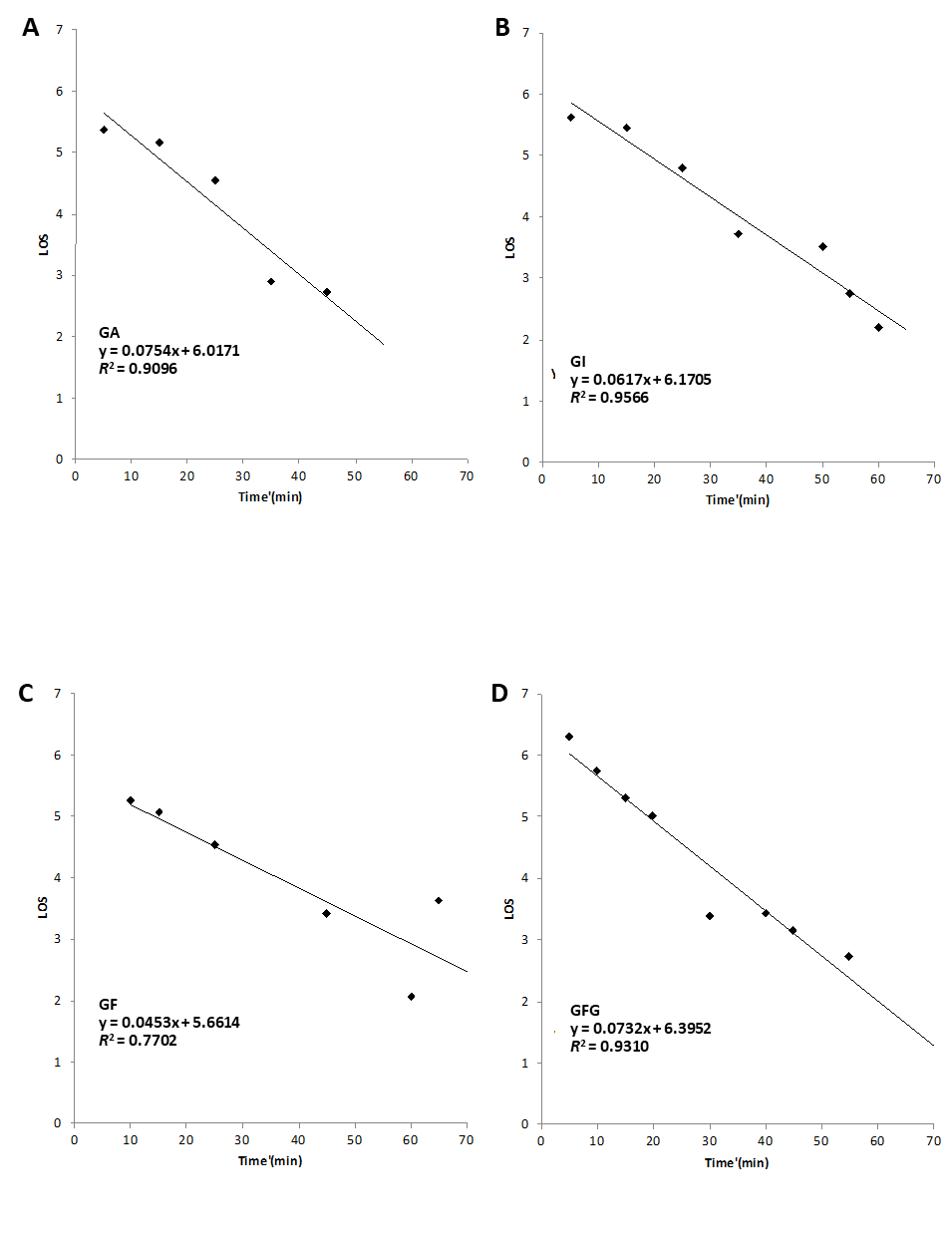
_**

**_
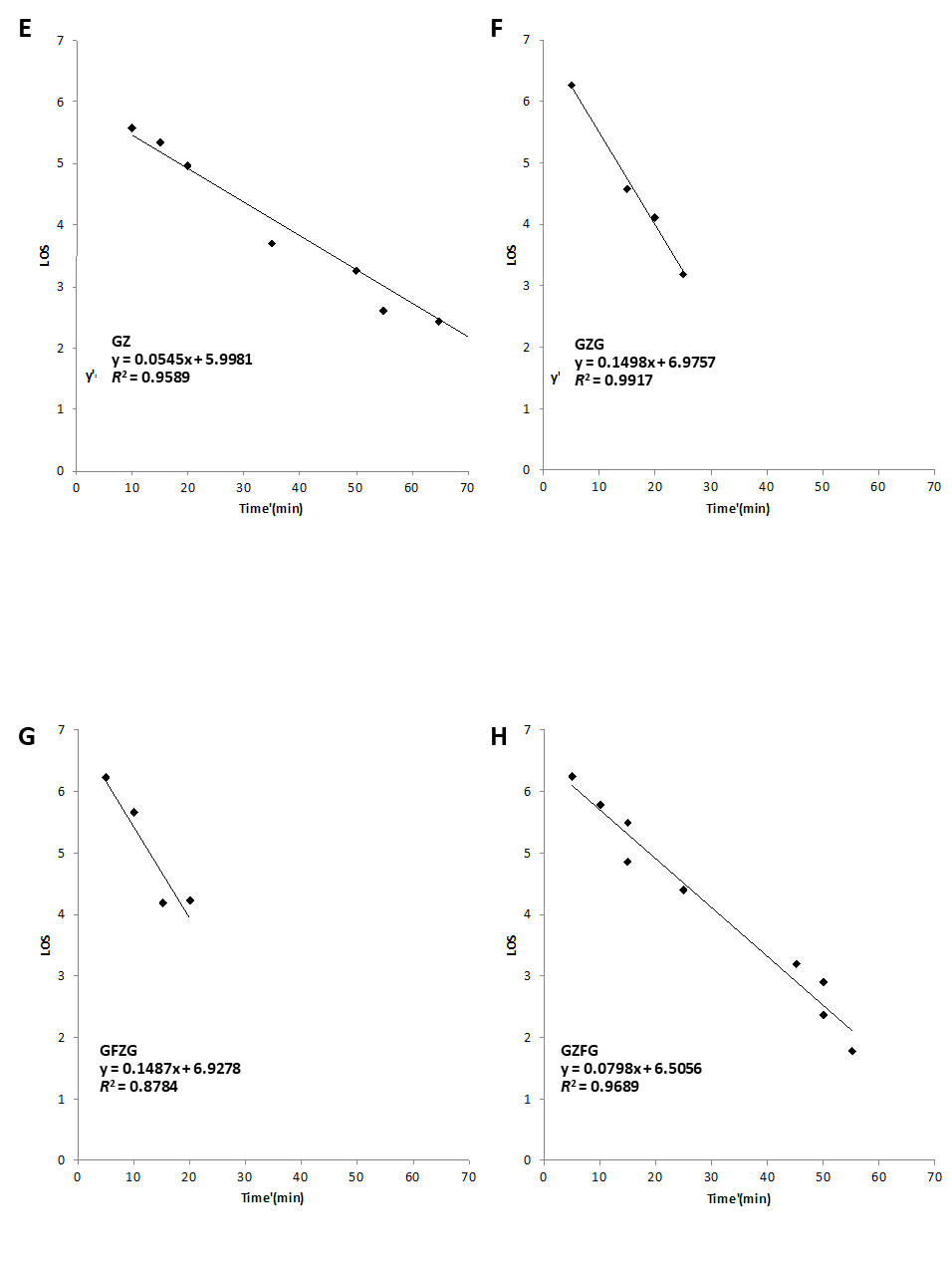
_**

**_
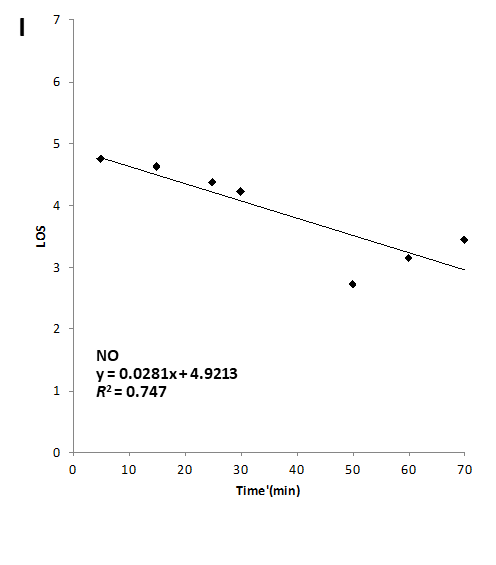
_**
